# Impact of Polystyrene Microplastics on Human Sperm Functionality: An In Vitro Study of Cytotoxicity, Genotoxicity and Fertility-Related Genes Expression

**DOI:** 10.3390/toxics13070605

**Published:** 2025-07-19

**Authors:** Filomena Mottola, Maria Carannante, Ilaria Palmieri, Lorenzo Ibello, Luigi Montano, Mariaceleste Pezzullo, Nicola Mosca, Nicoletta Potenza, Lucia Rocco

**Affiliations:** 1 Department of Environmental Biological and Pharmaceutical Sciences and Technologies, University of Campania Luigi Vanvitelli, 81100 Caserta, CE, Italy; filomena.mottola@unicampania.it (F.M.); maria.carannante@unicampania.it (M.C.); ilaria.palmieri@unicampania.it (I.P.); lorenzo.ibello@unicampania.it (L.I.); mariaceleste.pezzullo@unicampania.it (M.P.); nicola.mosca@unicampania.it (N.M.); nicoletta.potenza@unicampania.it (N.P.); 2 Andrology Unit and Service of Lifestyle Medicine in Uro-Andrology, Local Health Authority (ASL) Salerno, Coordination Unit of the Network for Environmental and Reproductive Health (EcoFood Fertility Project), S. Francesco di Assisi Hospital, 84020 Oliveto Citra, SA, Italy

**Keywords:** plastic micro fragments, male fertility, oxidative stress, DNA mutation, fertility gene expression

## Abstract

Polystyrene microplastics (PS-MPs) released in the environment reportedly affect the reproduction of various organisms, induced oxidative stress and apoptosis, resulting in altered sperm parameters. In this in vitro study, we tested the cytotoxicity and genotoxicity of PS-MPs by exposing human semen samples to PS-MPs levels (105 and 210 μg/mL) for 30–60–90 min. Semen parameters, genome stability, sperm DNA fragmentation (SDF) and reactive oxygen species (ROS) production were analyzed before and after exposure. Moreover, we also evaluated the expression level of spermatozoa-specific expressed genes essential for the fusion with oocyte (*DCST1*, *DCST2*, *IZUMO1*, *SPACA6*, *SOF1*, and *TMEM95*). After PS-MP exposure, semen concentration and morphology did not differ, while sperm vitality and motility decreased in a time-dependent manner. In addition, sperm agglutination was observed in the groups exposed to both PS-MPs concentrations tested. A time- and concentration-dependent reduction in genomic stability, as well as increased SDF and ROS production, was also observed. Moreover, all investigated transcripts were down-regulated after PS-MP exposure. Our results confirm the oxidative stress-mediated genotoxicity and cytotoxicity of PS-MPs on human spermatozoa. The sperm agglutination observed after treatment could be due to the aggregation of PS-MPs already adhered to the sperm membranes, hindering sperm movement and fertilizing capability. Interestingly, the downregulation of genes required for sperm–oocyte fusion, resulting from data on the in vitro experimental system, suggests that PS-MP exposure may have implications for sperm functionality. While these findings highlight potential mechanisms of sperm dysfunction, further investigations using in vivo models are needed to determine their broader biological implications. Possible environmental and working exposure to pollutants should be considered during the counselling for male infertility.

## 1. Introduction

Male fertility relies on the production of sperm, which must contain enough mature and functional sperm cells. Infertility can arise from disruptions in the normal processes of sperm production, maturation, motility, or release. The causes of male infertility are varied, ranging from genetic mutations to lifestyle factors, meaning it can be linked to both genetic and environmental influences [1,2]. The quality of the environment undeniably plays a significant role in human health. One of the major consequences of industrialization and technological development is the increased exposure to environmental toxins. Pollutants that impact reproductive organs are often referred to as endocrine disruptors (EDs). These substances can interfere with the hypothalamic–pituitary–gonadal axis by disrupting the synthesis of pituitary gonadotropins (FSH and LH), which in turn can adversely affect spermatogenesis [3,4]. Among the EDs causing growing concern, plastics have become some of the most studied in recent years due to their rapid proliferation driven by industrial development and human activities. The most significant threat from plastic pollution is its breakdown into smaller fragments, known as microplastics (MPs), with a diameter between 0.1 µm and 5 mm, and nanoplastics (NPs), which have a diameter of less than 0.1 µm [5].

Plastic fragments have been found in every corner of the planet, in both marine and terrestrial ecosystems, including oceans, rivers, air, drinking water, sediments, and food [6]. MPs can therefore be considered omnipresent, making the study of their impact on health essential. The human body encounters MPs through all possible routes of exposure, particularly the food chain [7,8,9]. Studies show that MPs smaller than 10 µm can move from the intestines to the lymphatic and circulatory systems, accumulating in tissues like the liver, kidneys, brain, lungs, intestines and reproductive system causing various types of toxicity [10,11]. Once internalized, MPs can cause oxidative stress (OS) and inflammatory responses, which are the key factors in MPs toxicity [12]. Recent research has increasingly focused on the toxic effects of polystyrene on the reproductive system as it is one of the most used plastic polymers for producing a wide range of products due to its high strength and chemical properties [13].

Studies, including one by Qiang and Cheng (2021) [14], have shown that polystyrene (PS) MPs (100 μg/L, 1000 μg/L) lead to higher levels of reactive oxygen species (ROS) and increased apoptosis in reproductive organs. Specifically, in zebrafish, exposure to PS-MPs caused elevated ROS levels, significant apoptosis in male testicles, and a notable reduction in the thickness of the testicular basal membrane. Similarly, studies on mammals have also shown harmful effects from PS-MPs. Jin et al. (2021) [15] exposed mice to MPs of various sizes and found that exposure led to testicular inflammation, disruption of the blood–testis barrier, decreased testosterone levels, and reduced sperm quality with an increased rate of abnormalities. Nevertheless, our recent results have shown that exposure to PS-MPs (50 μg/L) significantly reduce the viability and motility of sea urchin sperm and induce sperm DNA fragmentation through the production of ROS, negatively affecting the reproduction of this organism [16]. These experimental observations in aquatic species and rodents, combined with epidemiological evidence of declining sperm quantity and quality worldwide, highlight a serious issue for the future of the human species as the male reproductive system appears to be particularly sensitive to environmental stressors [17].

Although the health effects on exposed organisms are well-documented, the behavior and mechanism of action of MPs remain not fully understood. In a sea urchin study [16], exposure to PS-MPs (50 μg/L) resulted in sperm agglutination. Due to their properties and the surrounding environment, MPs adhere to the sperm cell membranes, leading to the formation of sperm aggregates that limit sperm motility. Recent studies have shown that the intrinsic physico-chemical properties of MPs endow them with aggregation abilities, which are generally influenced by the ionic strength and electrolyte valence of the surrounding medium [18]. This aspect, combined with the induction of OS, could partially explain their reproductive toxicological mechanism.

It is known that environmental EDs may impact biological processes by altering the DNA methylation and affecting the expression of genes associated with steroidogenesis [19,20].

A systematic review of the literature has shown that exposure to MPs increases the expression of genes and proteins involved in apoptosis and inflammatory processes, such as Bik, Bax, p-JNK, p-p38 MAPK, and caspase 3. At the same time, it decreases the expression of genes and proteins associated with gonadal function and cellular barriers, such as ABP, PLZF, Bcl2, occludin, N-cadherin, and claudin-11. These findings indicate that MPs and NPs can negatively affect gonadal health [21].

While the effects of pollution on genes related to apoptosis and inflammation are the focus of numerous studies, particularly concerning fertility, the impact on genes responsible for the mechanism of sperm–oocyte fusion remains largely underexplored.

The genes involved in the fusion of sperm with the oocyte play a crucial role in reproduction, as they regulate the essential molecular processes required for fertilization. Alterations in the expression of these genes can compromise the ability of sperm to penetrate the oocyte, leading to fertility issues [22]. Investigating how the expression of these genes changes in response to EDs represents an emerging and innovative field of research. Understanding the responses of these genes to MPs provides new insights into the molecular mechanisms by which these pollutants can impact fertility.

The aim of this research was to evaluate, in vitro, the cytotoxic and genotoxic effects of PS-MPs (105 and 210 μg/mL) for 30–60–90 min of exposure on human sperm, highlighting any alterations in semen parameters, ROS production, and genetic material alterations in terms of genome instability and sperm DNA fragmentation (SDF). Next, we decided to explore the impact of PS-MP treatment on the expression levels of genes involved in male fertility, specifically those required for sperm–oocyte fusion, a key step for a successful fertilization and emerging as a multifactorial and very complex process. An inspection of literature led us to select and evaluate the expression level of the following genes: (i) *IZUMO1*, long been known to be essential for sperm–egg fusion, by forming a 1:1 complex with oocyte Juno (also known as IZUMO1R, IZUMO1 receptor) [23,24,25,26]. (ii) *DCST1* (dendrocyte expressed seven transmembrane protein domain-containing 1) and (iii) *DCST2* (dendrocyte expressed seven transmembrane protein domain-containing 2), the evolutionarily conserved factors from nematode or fruit fly to human involved in gamete recognition and fusion and more recently discovered to be essential for sperm–egg fusion in mice and fish [27,28]. (iv) *SOF1* (genes, sperm−oocyte fusion required 1), (v) *SPACA6* (sperm acrosome associated 6) and (vi)*TMEM95* (transmembrane protein 95), three testis-enriched proteins essential for sperm−oocyte fusion in mice; although rarely conserved in animal species, such as flies, frogs, and birds, the three relative genes are widely conserved in mammals, such as rodents, livestock (e.g., pig and cow), and primates, including human, similarly to phylogenetic tree of *IZUMO1* and thus emerging as fusion-related genes in gametes during evolution [22,29,30,31].

This research could lead to new strategies to prevent or mitigate the effects of endocrine disruptors on reproductive health, improving the diagnosis and treatment of fertility disorders. Moreover, understanding the mechanisms of sperm–oocyte fusion in response to environmental factors can contribute to the development of targeted interventions to preserve human fertility in an increasingly contaminated world.

## 2. Materials and Methods

### 2.1. Chemical

The study used the previously described polystyrene microplastics [16] with a diameter of 1.0 μm, a specific gravity of 1.05 g/cm^3^, and a solid content of 2% by weight. The MPs, provided as a 21,000 mg/L concentrated aqueous solution containing about 3.43 × 10^10^ particles per mL, were obtained from Sigma-Aldrich (St. Louis, MO, USA; product code: 72.938; batch number: BCCH6977; analytical standard purity).

### 2.2. Sperm Collection and Exposure

The men recruited for this study were referred to the Reproduction Biology Laboratory at the University “L. Vanvitelli” in Caserta, Italy, for routine semen analysis. They were aged between 27 and 43 years and were not undergoing treatment with any drugs or antioxidants. Specifically, seminal fluids from 10 man were selected whose parameters: sperm concentration, viability, motility, and normal morphology, after sperm analysis, met the criteria of the World Health Organization [32] and were therefore defined as normospermic (normal condition of seminal fluid) (Table 1). The percentage of morphologically normal spermatozoa was evaluated using Test-simplets^®^ stained slides (Origio; Cooper Surgical, Inc., Måløv, Denmark) under an optical microscope (Carl Zeiss, Germany) at 40× magnification. The eosin test was used to assess sperm viability (Sigma-Aldrich, St. Louis, MO, USA, CAS No. 15086-94-9;) [33], while the evaluation of sperm motility was performed by a Makler counting chamber as recently described by Mottola et al. (2024) [16].

A minimum of 200 spermatozoa from each experimental group were examined across five different observation fields on three separate slides.

Samples were purified by discontinuous density gradient centrifugation, using a 45% and 90% double density gradient to recover motile and viable spermatozoa. Then, the samples were diluted with HEPES-buffered minimum essential medium (MEM) (Sigma Aldrich^®^) to a final concentration of 7 × 10^6^/mL. Subsequently, they were exposed in vitro to two concentrations of PS-MPs, 105 μg/mL and 210 μg/mL, selected based on previous data, for 30, 60 and 90 min. We selected a concentration of 105 μg/mL based on previous in vitro studies that used similar levels to analyze cellular responses [34,35,36,37]. This dose allows the observation of specific effects such as DNA damage, ROS production, and apoptosis, considering that the in vitro environment, unlike in vivo, lacks compensatory physiological mechanisms. To simulate extreme contamination conditions, we also tested a higher concentration of 210 μg/mL, consistent with studies describing highly polluted environments [38,39,40,41], in order to evaluate the maximum toxic impact. To obtain the desired concentrations, the PS-MPs solution was diluted in 1× phosphate-buffered saline (PBS). Untreated samples were exposed to an equal volume of PBS 1× and served as a negative control, while samples treated with 1.5 mM of hydrogen peroxide (H_2_O_2_), were utilized as a positive control [16]. After exposure, the samples were centrifuged for 5 min at 1500 rpm and the pellet was suspended in 500 μL of 1× phosphate-buffered saline (PBS), then, the semen parameters were re-evaluated and all cytotoxicity and genotoxicity test were performed in triplicate. For gene expression analysis, the immature sperm cells, including spermatogonia, spermatocytes and spermatids, recovered from the supernatant after gradient centrifugation were exposed for 30, 60 and 90 min to 210 μg/mL PS-MPs. Afterward, the sperm cells were recovered after centrifugation for 5 min at 1500 rpm and the pellet was suspended in Phenol/guanidine-based QIAzol Lysis Reagent (Qiagen, Hilden, Germany) for subsequent RNA purification.

### 2.3. Sperm DNA Fragmentation

The TUNEL assay was performed using an In Situ Cell Death Detection Kit (Roche Diagnostics^®^, Basel, Switzerland). In this assay, spermatozoa with double-strand DNA breaks emit green fluorescence due to the fluorescein isothiocyanate (FITC) used as marker. To differentiate fragmented DNA from intact DNA, the sperm cells were also stained blue with the fluorochrome DAPI (4,6-diamino-2-phenylindole). According to Mottola et al. (2022) [42], after fixation in 4% paraformaldehyde for 1 h, each slide containing sperm samples was permeabilized with a solution of sodium citrate, distilled water, and Triton X-100 on ice for 2 min. Subsequently, 5 µL of enzyme solution and 45 µL of labeling solution were applied to each slide and incubated for 60 min at 37 °C in a humidified chamber. The slides were then examined with a fluorescence microscope (Nikon Eclipse E-600, Tokyo, Japan) using BP 330–380 nm and LP 420 nm filters. The sperm DNA fragmentation percentage (SDF, %) was determined by calculating the proportion of green sperm relative to the total number of sperm cells. At least 250 spermatozoa from each experimental group were analyzed.

### 2.4. RAPD-PCR and GTS

Prior to performing the RAPD-PCR technique, DNA was isolated from sperm cells using the High Purity PCR Template Preparation Kit (ROCHE Diagnostics^®^, Basel, Switzerland) to obtain a highly pure sample. The RAPD-PCR was performed using a ready-to-use reaction mix (Master PCR FastStart™ (ROCHE, Basel, Switzerland, REF 4710436001), to which a specific primer 6 (5′CCCGTCAGCA3′), 40 ng of sperm DNA and enough high pure water were added to reach a final volume of 25 µL. This was subjected to follows cyclic amplification programme in a thermal cycler: initial denaturation step at 94 °C for 2 min and 45 cycles of 95 °C for 1 min, annealing at 36 °C for 1 min and extension at 72 °C for 1 min. Finally, the amplified products are separated by agarose gel electrophoresis (1.5%) and analyzed by a ChemiDoc Gel Imaging System (Bio-Rad, Hercules, CA, USA) to determine the percentage of genomic template stability (GTS, %) using the following formula: (1 − a/n) × 100, where “a” represents the average number of polymorphic bands observed in each exposed sample, and “n” denotes the total number of bands present in the untreated cells. Polymorphism in the RAPD-PCR profiles was identified by noting the absence of certain bands and the emergence of new bands compared to the control, which was set at 100% [43].

### 2.5. NitroBlue Tetrazolium Chloride Test (NBT)

The NitroBlue Tetrazolium Chloride (NBT) test measures superoxide anion production in cells. NBT, a yellow water-soluble compound, turns into an insoluble blue formazan crystal when it reacts with superoxide anions. Each experimental group (spermatozoa exposed to plastic fragments, hydrogen peroxide, and untreated controls) was diluted in 1 mL of nitroblue tetrazolium (NBT) solution (Sigma-Aldrich, St. Louis, MO, USA, CAS N. 298–83-9) and incubated at 37 °C for 15 min. After centrifugation at 2000 rpm for 1 min to remove unreacted NBT, 1 mL of 1× PBS was added. The samples were then centrifuged again at 1500 rpm for 5 min. This washing procedure was repeated three times to ensure thorough purification of the cell samples.

At the end of the final wash, the supernatant was discarded, and the pellet was resuspended in 200 µL of 1× PBS. From this suspension, 100 µL were placed on a slide. After the slide air-dried, it was fixed with 100% methanol (VWR Chemicals, Radnor, PA, USA, CAS 67–56-1) for 1 min and then rinsed with highly purified water. Safranin (Sigma-Aldrich, St. Louis, MO, USA, CAS N. 477–73-6) was used as a counterstain to enhance cell visibility. The slide was air-dried and examined under optical microscope at 40× magnification.

The result of this test is expressed as the percentage of reactive oxygen species (ROS, %), calculated as the ratio of cells showing ROS to the total number of cells, multiplied by 100. Thus, ROS, % is determined as the ratio of sperm cells exhibiting blue formazan crystal reaction to the total number of sperm cells. As for TUNEL Assay, 250 spermatozoa from each experimental group were analyzed.

### 2.6. RNA Purification and Real-Time PCR Analyses

Total RNA was extracted from cells using the RNeasy mini kit according to manufacturer’s protocol (Qiagen, Hilden, Germany).

For quantification of the transcripts, total RNA was retrotranscribed using the SensiFAST cDNA Synthesis kit (Bioline, London, UK).

Then, standard SYBR Green Real-time qPCR assays were performed with the primers, designed by Primer-Blast (https://www.ncbi.nlm.nih.gov/tools/primer-blast/) and Primer3 (https://primer3.ut.ee/, Tartu, Estonia), (access date: 17 March 2025) GAPDH was selected as reference genes due to its stable expression across the different experimental conditions and its high detectability, with a Ct value consistently around 20 (Table 2). The expression levels of the different transcripts were normalized to the reference one by using the 2^−ΔCt^ method [44,45].

### 2.7. Statistical Analysis

Differences in the percentage of seminal parameters, including SDF, ROS, and GTS, %, among experimental groups were reported as mean ± SD. Statistical analysis was performed using the U test (Mann–Whitney test) and ANOVA (analysis of variances) via GraphPad Prism 10 (GraphPad Software Inc., San Diego, CA, USA).

## 3. Results

### 3.1. Sperm Analysis

**Figure 1:**
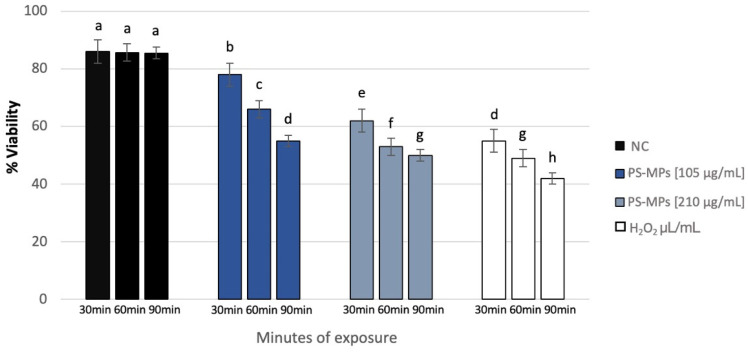
Percentage of viability (ordinate) in human sperm cells after different exposure minutes (abscissa) to 105 μg/mL PS-MPs and 210 μg/mL PS-MPs. Different letters indicate statistically significant differences (ANOVA). *p* ≤ 0.05.

**Figure 2:**
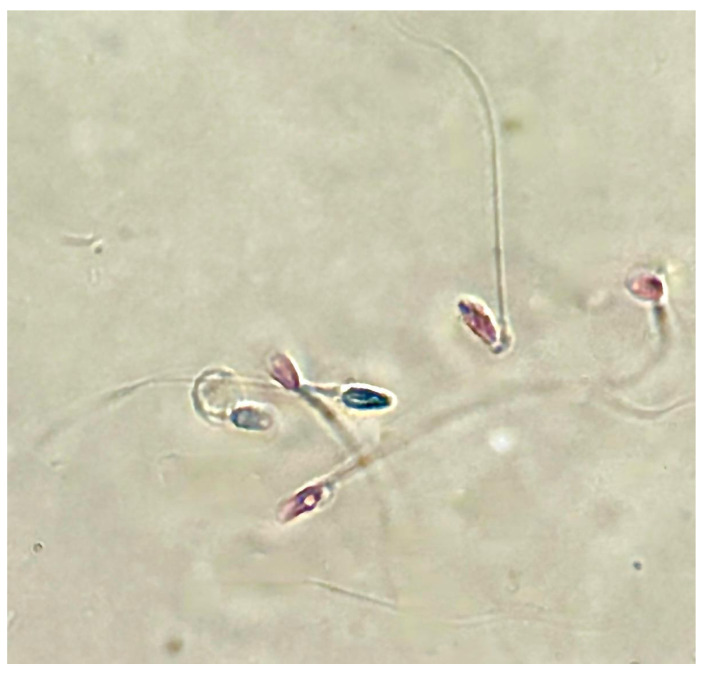
Representative image obtained by Nikon Eclipse E600 microscope (Tokyo, Japan) at 60× magnification of sperm viability after treatment with 210 μg/mL PS-MPs for 90 min. Viable cells are translucent, while non-viable cells are stained.

**Figure 3:**
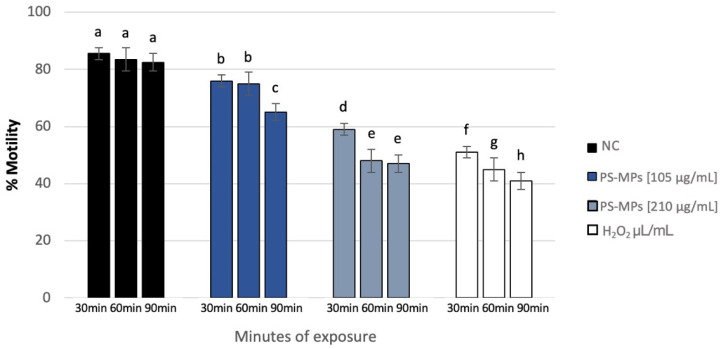
Percentage of motility (ordinate) in human sperm cells after different exposure minutes (abscissa) to 105 μg/mL PS-MPs and 210 μg/mL PS-MPs. Different letters indicate statistically significant differences (ANOVA). *p* ≤ 0.05.

Sperm analysis did not highlight changes in terms of number and morphology of the spermatozoa in any of the tested concentrations of PS-MPs, while there was a decrease in viability and motility following treatment with PS-MPs in a timely manner-dependent, especially at the highest concentration tested. Specifically, Treatment with 105 µg/mL PS-MPs resulted in a significant reduction in sperm cell viability after 60 and 90 min of exposure, with values of 66% ± 2 and 55% ± 4, respectively, compared to the negative control (86% ± 4) and the positive control (43% ± 3). A significant decrease in motility was observed only at the longest exposure time (90 min), with a value of 65% ± 2 compared to the untreated sample (83% ± 3) Following 30 min of exposure to 210 µg/mL PS-MPs, sperm viability was 62% ± 2; after 60 min, it dropped to 53% ± 1, and after 90 min, it further decreased to 50% ± 3, compared to the negative control (86% ± 4) (Figure 1). A representative image of sperm viability following treatment with 210 µg/mL PS-MPs for 90 min is shown below as Figure 2. In contrast, sperm motility was 59% ± 5, 48% ± 4, and 47% ± 2 after 30, 60, and 90 min of treatment, respectively, which was Statistically lower than the negative control (82% ± 2) (Figure 3).

**Figure 4:**
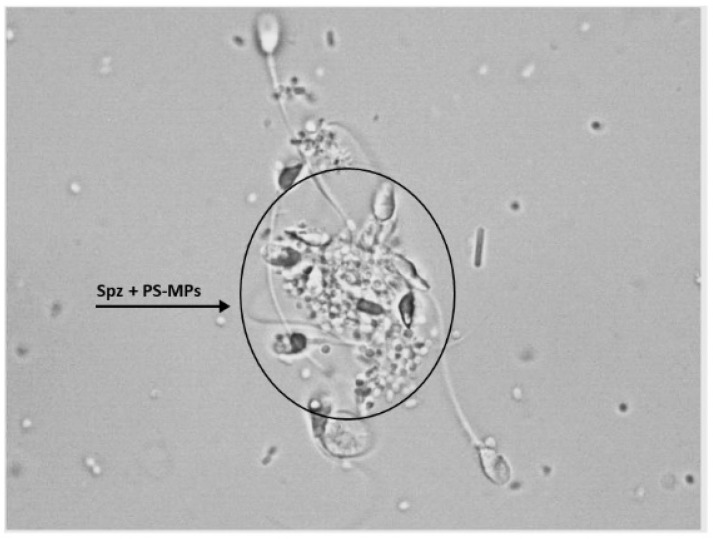
Representative image obtained by Nikon Eclipse E-600 microscope (Tokyo, Japan) at 100× magnification showing human sperm agglutination after a 90 min treatment with 210 µg/mL of PS-MPs. The presence of microparticles between sperm cells is highlighted by the circle.

Furthermore, following treatment with PS-MPs, agglutination of spermatozoa was observed at all three exposure times at both concentrations (Figure 4).

### 3.2. TUNEL Assay

**Figure 5:**
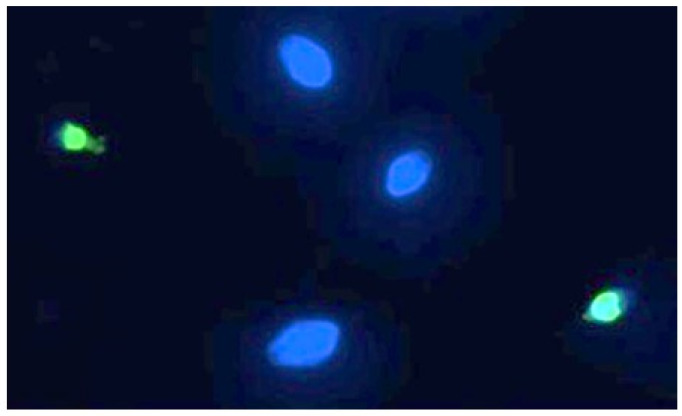
Representative image of human spermatozoa after 90 min of treatment with 210 µg/mL PS-MPs, obtained using the TUNEL technique. The image was acquired by Nikon Eclipse E-600 microscope (Tokyo, Japan) at 100× magnification Spermatozoa with intact DNA are shown in blue (DAPI filter), while those with fragmented DNA are shown in green (FITC filter).

Sperm DNA fragmentation, of which a representative image can be seen in Figure 5, is statistically significant compared to the controls following exposure to 90 min of PS-MPs.

**Figure 6:**
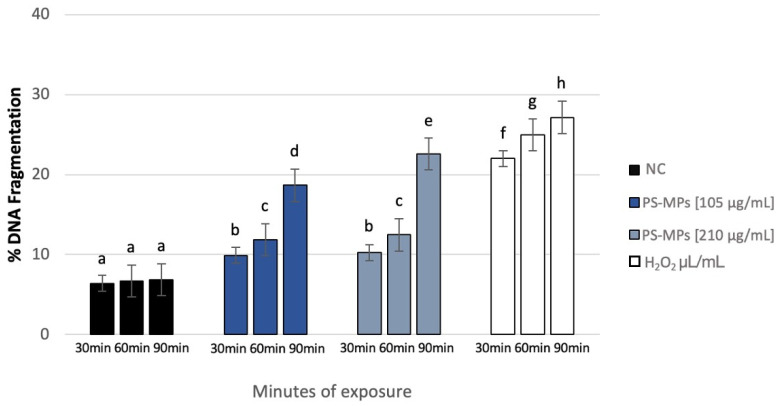
DNA fragmentation index (ordinate) in human sperm cells after different exposure minutes (abscissa) to 105 μg/mL PS-MPs and 210 μg/mL PS-MPs. Different letters indicate statistically significant differences (ANOVA). *p* ≤ 0.05.

After treatment with 105 µg/mL PS-MPs, SDF (%) at the maximum exposure time (90 min) reached 18.66% ± 4, compared to the negative control (6.86% ± 1). SDF values were 9.9% ± 2 and 11.84% ± 1 after 30 and 60 min of treatment, respectively. Similarly, after treatment with 210 µg/mL PS-MPs, SDF reached 22.6% ± 4 at 90 min, 12.46% ± 1 at 60 min, and 10.25% ± 2 at 30 min (Figure 6).

### 3.3. RAPD-PCR Profile

In comparing the RAPD-PCR results to the negative control, we observed notable variations in polymorphic bands for both tested concentrations and all exposure times. RAPD-PCR profile on control samples showed distinct bands at 200, 300, 400, 500, 550, 650, 700, 800, 980, and 3000 bp. In contrast, notable variations were observed for both tested concentrations and all exposure times. After 30 min of exposure to 105 μg/mL PS-MPs we noted the disappearance of a 290 bp band. After 60 min, there was not only a loss of bands at 500, 700, and 1000 bp but also the emergence of a new band at 200 bp. Following 90 min of 105 μg/mL PS-MPs treatment, bands at 290, 400, 550, and 1100 bp were no longer present, while a 350 bp band. For the higher concentration of 210 μg/mL we noted after 30 min of exposure that the bands at 290 and 350 bp were absent, and a new band at 690 bp was detected. 60 min of exposure to 210 μg/mL PS-MPs induced the disappearance of bands at 350, 500, 700, 1000, and 1100 bp. After 90 min, bands at 290, 320, 700, and 1000 bp were no longer visible, while new bands at 200 and 300 bp emerged (refer to Table 3 for details).

### 3.4. Genomic Template Stability

**Figure 7:**
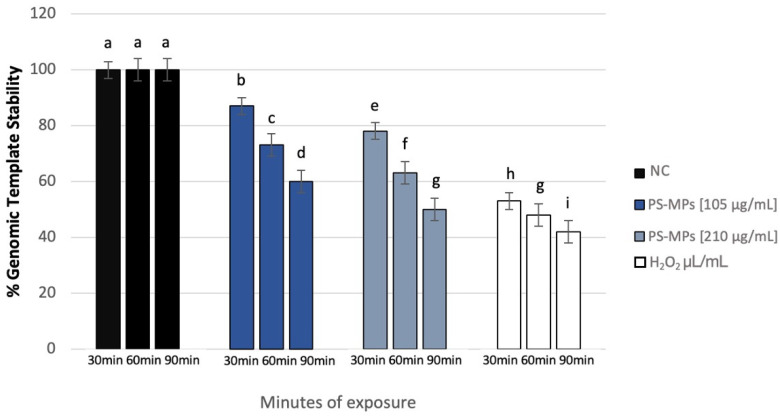
Changes in the percentage of Genome Template Stability in human sperm DNA (ordinate) after different exposure minutes (abscissa) to 105 μg/mL PS-MPs and 210 μg/mL PS-MPs. Different letters indicate statistically significant differences (ANOVA). *p* ≤ 0.05.

The polymorphic profiles obtained through the RAPD-PCR technique were utilized to calculate the percentage of template stability (%GTS) following treatment with PS-MPs. A time-dependent decrease in genomic stability was noted at the highest concentration tested. Specifically, at a concentration of 105 μg/mL PS-MPs, a reduction in genomic stability of 13% ± 5 was observed after 30 min of exposure compared to the negative control. This reduction became statistically significant at 60 and 90 min, with decreases of 27% ± 4 and 40% ± 2, respectively. At the concentration of 210 μg/mL PS-MPs, a statistically significant reduction in genomic stability was evident, with a decrease of 22% ± 5 after just 30 min of exposure compared to the negative control. This reduction increased to 37% ± 4 after 60 min and 50% ± 2 after 90 min of treatment (Figure 7).

### 3.5. ROS Detection

**Figure 8:**
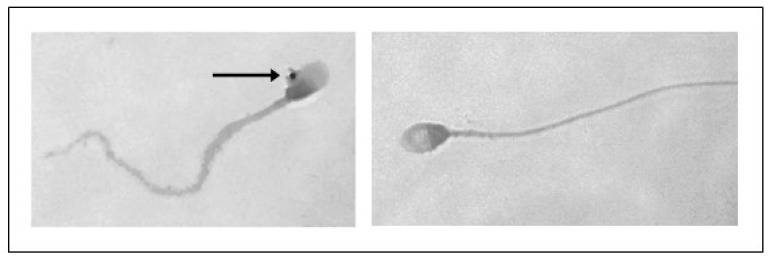
Representative image obtained by ZEISS Germany AXIOSKOP microscope (Oberkochen, Germany) at 100× magnification of reactive oxygen species (ROS) in human spermatozoa, highlighted by NBT reaction (indicated by arrows), following 90 min of treatment with 210 µg/mL PS-MPs.

**Figure 9:**
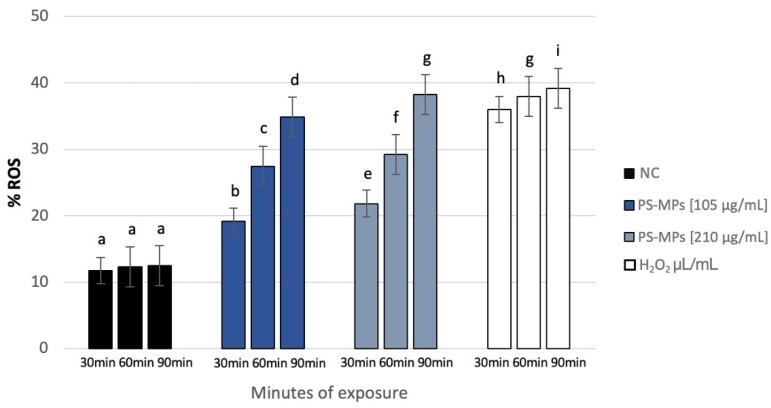
Percentage of ROS (ordinate) in human sperm cells after different exposure minutes (abscissa) to 105 μg/mL PS-MPs and 210 μg/mL PS-MPs. Different letters indicate statistically significant differences (ANOVA). *p* ≤ 0.05.

NBT analysis revealed a statistically significant increase in intracellular ROS in human spermatozoa treated with PS-MPs at all assessed time points, as shown in the representative image (Figure 8), particularly at the highest concentration of PS-MPs. The %ROS after treatment with 105 µg/mL PS-MPs was 19.14% ± 1.06 after 30 min, 27.49% ± 1.44 after 60 min, and 34.88% ± 1.47 after 90 min, compared to the negative control (12.1 ± 0.21). Furthermore, the percentage of formazan-positive spermatozoa increased from 21.85% ± 2.71 after 30 min of exposure to 210 µg/mL PS-MPs, to 29.22% ± 1.41 after 60 min, and 38.24% ± 1.19 after 90 min. This trend suggests a dose–response relationship between exposure to 210 µg/mL PS-MPs and oxidative damage (Figure 9).

### 3.6. Expression of Sperm–Egg Fusion Genes

**Figure 10:**
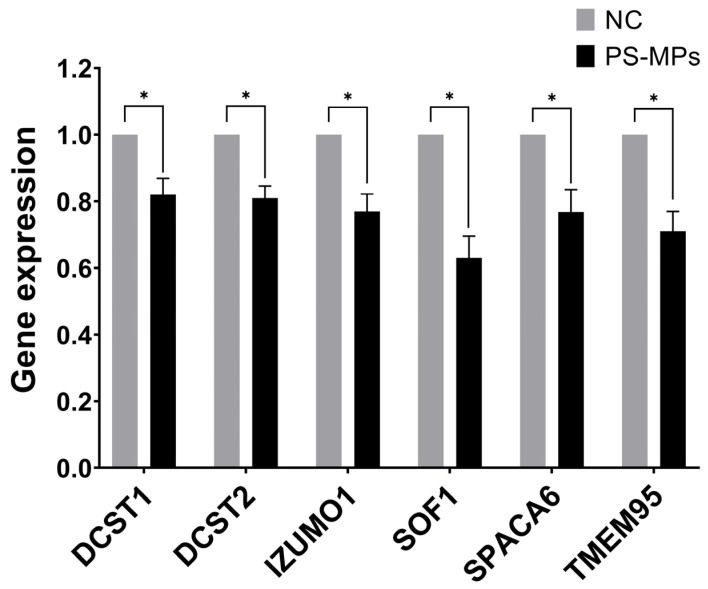
Effect of PS-MPs on the expression of genes involved in sperm ability to fuse with oocytes. The expression level of the indicated genes was evaluated by RT-QPCR on RNA purified from non-treated negative control (NC) or PS-MP-treated immature sperm cells for 90 min at 210 µg/mL. The values are reported as fold mean (2^−ΔΔCt^) relative to NC. * *p* < 0.05.

We set up the optimal primers for the human genes homologous to those consistently reported as essential for the spermatozoa ability of egg-fusion, and RT-qPCR conditions to assess simultaneously their expression levels in human semen samples and those exposed at 210 μg/mL of PS-MPs for 90 min. We found that all the tested genes have been down-regulated by PS-MPs treatment in comparison to the negative control, ranging from 37% and 29% inhibition for *SOF1* and *TME95*, respectively, to similar percentage of about 24% for *IZUMO1* and *SPACA6*, to about 20% reduction for *DCST1* and *DCST2* (Figure 10). The observed down-regulation suggests that PS-MPs exposure could result in impaired functionality of the sperm–oocyte fusion machinery at the molecular level. However, further research, including in vivo studies, is needed to confirm these effects and to understand the broader implications for reproductive health.

## 4. Discussion and Conclusions

Over the past twenty years, male fertility has significantly declined worldwide, primarily due to a reduction in sperm count attributed to exposure to environmental toxins. Among these contaminants, plastic, particularly polystyrene (PS), poses a significant threat to human reproductive and environment [46] as it is widely used for many products, because of its resistance, flexibility and low cost [13].

While the primary cause of declining fertility remains unclear, evidence from studies on marine and land animals highlights the significant threat posed by PS-MPs to reproduction as it can enter into the organisms through inhalation, oral exposure, and skin absorption, and once inside, they can cross cellular membranes, causing inflammation, oxidative stress, and DNA damage in sperm, leading to alteration in seminal parameters [47,48].

In a study by Qiang and Cheng (2021) [14] on zebrafish, exposure to PS-MPs (100 μg/L) led to a notable increase in ROS in both male and female gonads, with high levels of apoptosis and a significant reduction in testis basement membrane thickness.

Very limited research data exist on how and whether exposure to PS-MPs damages the mammalian reproductive system. As already mentioned, the study conducted by Jin and co-workers (2021) [15] on mice showed that exposure to PS-MPs (10 mg/mL) induced testicular inflammation and destruction of the blood-testicular barrier, thus having negative effects on spermatogenesis; in addition, studies on rodents have shown that PS-MPs (50 µg/mL, 100 µg/mL, 200 µg/mL, 400 µg/mL and 800 µg/mL) have harmful effects on the male gonads, these effects include degeneration of the seminiferous tubule epithelium, thinning of the testicular basement membrane, death of Sertoli cells, disruption of the blood-testis barrier, formation of malformed sperm [34,49,50,51].

The reactive oxygen species generate by PS-MPs can, in turn, cause damage to DNA and sperm cell membranes, by reducing antioxidant enzymes (CAT, SOD, POD) concentration, as well as sperm number, motility and viability [52]. Additionally, PS-MPs have been found to act as endocrine disruptors interfering with the hypothalamic-pituitary-testicular axis and altering the levels of essential hormones like testosterone, FSH, and LH, which are crucial for spermatogenesis [53].

In this study, to assess the effects on human male reproduction, we tested the cytotoxicity and genotoxicity of two concentrations of PS-MPs (105 and 210 μg/mL) on human sperm in vitro for 30, 60 and 90 min, focusing on evaluating the changes in semen parameters induced by MPs, as well as genome stability, reactive oxygen species production, and the expression levels of genes involved in sperm–oocyte fusion; this aspect has been considered for the first time in mammalian reproductive genotoxicity studies.

Microplastic concentrations of 105–210 μg/mL are not currently observed in real-world environments, but their use in vitro has a well-defined scientific rationale. These levels are often employed to explore dose–response relationships and identify critical thresholds beyond which toxic effects, such as cellular damage, inflammation, or genetic alterations, begin to manifest. This approach is common in toxicology and allows for a deeper understanding of the mechanisms of microplastic action Moreover, elevated concentrations can simulate extreme scenarios, such as severe future pollution or specific highly contaminated microenvironments, like sediments near industrial discharges. Another advantage of these concentrations is their ability to amplify potential biological effects, facilitating the detection of subtle changes that might otherwise go unnoticed [34,35,36,38,39,40,41]. However, it is important to note that such high concentrations may not directly reflect typical environmental exposure levels. Therefore, it is crucial to complement these studies with experiments conducted at lower concentrations if the aim is to extrapolate in vitro findings to ecological or physiological scenarios.

In this study, we used three complementary approaches to investigate the genomic stability of spermatozoa exposed to polystyrene microplastics (PS-MPs): the TUNEL assay, the RAPD-PCR profile, and genomic template stability (GTS). Although all three explore a common theme, each provides unique and complementary information. The TUNEL assay provides a direct indication of DNA fragmentation, a key marker of apoptosis and cellular damage, with the resulting data reflecting the level of genomic damage closely linked to programmed cell death mechanisms [54]. On the other hand, the RAPD-PCR technique focuses on random changes in the DNA amplification pattern, highlighting variations at the global genomic structure level. This method offers a broader measure of genomic stability, allowing the detection of potentially more subtle yet significant alterations [55]. Genomic Template Stability (GTS), derived from RAPD-PCR data, quantifies genetic variations in a numerical and comparative way, providing a global parameter that facilitates comparisons between different experimental groups. This strategy allowed us to address the issue of genomic stability from multiple perspectives, thereby offering a detailed and multidimensional analysis of genome stability [56].

Recently, some researchers have suggested that exposure to MPs might impair male fertility by reducing the ability of spermatozoa to fertilize eggs. However, the researchers propose that the mechanism of action involves the alteration of mitochondrial structure and function [34], while Sussarellu and co-workers (2016) [57], in oysters exposed to PS-MPs, showed a reduced ability of sperm to fertilize oocytes due to lower sperm motility.

In the present research, exposure to PS-MPs led to a time-dependent decrease in sperm vitality and motility, while semen concentration and morphology remained stable. This suggests that in vitro exposure to PS-MPs primarily affect sperm functionality rather than their basic morphology or quantity. In fact, there is no clear evidence that a contaminant can alter the morphology of spermatozoa after ejaculation. While exposure to contaminants can interfere with the stages of germ cell development in the testes, causing morphological abnormalities in mature spermatozoa [15]. Considering that MPs can reach the testicles and end up in the seminal fluid [10,58], it cannot be ruled out that in vivo exposure might also alter the spermatozoa morphology. Similarly, the number of spermatozoa decreases is typically due to DNA fragmentation-mediated apoptosis, which ultimately leads to complete cell degeneration. Although, our study evidenced an increased sperm DNA fragmentation, DNA instability and mortality, particularly at higher concentrations, this contradicts the lack of observed reduction in sperm count. Damage to sperm DNA induct by EDs is consistent with the literature as can occur, along with alterations in chromatin architecture, mainly due to DNA fragmentation, which can affect subsequent embryonic development [3,59], but it should be noted that DNA fragmentation is merely an intermediate stage of apoptosis, so it cannot be ruled out that an in vivo exposure might also result in a reduction in sperm concentration. To support this hypothesis, the time-dependent nature of these effects highlighted by our experiments, indicates that longer exposure could exacerbate the damage. Regardless, the observed reduction in sperm motility caused by MPs is a certain and concerning finding for male reproductive health with oxidative stress serving a key mechanism driving sperm dysfunction. Increased ROS production correlated with the duration and concentration of PS-MPs exposure suggests that they can produce an inflammatory-oxidative environment, resulting in the creation of genotoxic oxidants [60]. Among the harmful effects of oxidative stress, sperm motility is particularly compromised, as polyunsaturated fatty acids in their membrane facilitate ROS attacks, leading to lipid peroxidation. This process alters sperm membranes and mitochondria, reducing the energy production capacity needed for movement [61].

Furthermore, our results showed that the alteration in movement is also caused by the agglutination of sperm in the presence of PS-MPs, as previously observed in sea urchin models [16]. Recent studies have shown that the intrinsic chemical-physical properties of microplastics confer an aggregation capacity, typically influenced by the ionic strength and electrolyte valence of the surrounding medium [18]. As already reported in our previous study [16], sperm agglutination occurs due to the presence of anti-sperm antibodies in seminal fluid. In this research, agglutination was induced by PS-MPs which, due to their properties, adhered to the sperm cell membrane, creating binding bridges with other microplastics attached to other sperm cells, leading to the formation of sperm aggregates, a condition not observed in the negative and positive control groups. However, it is important to note that, to date, the only evidence of MPs in seminal fluid refers to mere traces [10]. The concentration used in our study was intentionally chosen to produce an observable effect. Therefore, we do not expect such an amount to accumulate in the testes to the extent of causing agglutination that blocks sperm motility. While these findings are based on in vitro experiments, they offer valuable preliminary insights into the potential interactions and effects of microplastics, helping to guide further in vivo investigations needed to fully understand their impact.

In this regard, it would be crucial to include a control with particles of similar size to determine whether the observed toxic effects are truly attributable to polystyrene microplastics and not to other material characteristics. However, a significant limitation lies in the difficulty of obtaining non-toxic particles of the same size. This is because size and surface properties significantly influence toxicity [62].

An alternative option could be the use of polystyrene particles with surface modifications specifically designed to reduce interaction with cells. However, such modifications have not yet been developed: existing literature primarily focuses on modifications aimed at increasing cellular affinity rather than decreasing it [63,64,65]. Once available, these approaches would allow a clearer understanding of the specific roles of size, surface chemistry, and material composition in the observed genotoxicity, thereby contributing to a more precise understanding of the effects of microplastics.

An even more interesting finding emerging from our studies is the ability of MPs to induce a downregulation of genes involved in the mechanism of sperm–oocyte fusion. This is the first study investigating the potential impact of pollution, and in particular of MPs, on a selected panel of genes expressed in spermatozoa samples and required for fertilization, also setting up a qPCR-based tool for further investigations. In fact, so far only one paper reported a significant reduction in IZUMO1 protein content in sperm samples of young men exposed to toxic environmental contaminants [66]. IZUMO1, DCST1, DCST2, SPACA6, SOF1, and TMEM95 are key proteins that play a fundamental role in the process of sperm–oocyte fusion during fertilization in mammals. IZUMO1 is a key protein on sperm that binds to JUNO, a protein on the oocyte, to enable membrane fusion and initiate fertilization. DCST1 and DCST2 are also essential proteins for sperm–oocyte fusion, as genetic experiments have shown that the lack of these proteins blocks fertilization [27]. SPACA6, initially located in the acrosomal cap of the sperm and later in the equatorial segment, is crucial for membrane fusion, with mechanisms regulated differently compared to IZUMO1, DCST1, and DCST2, though not fully defined. *SOF1* is another essential gene for gamete fusion; it undergoes modifications during sperm maturation that suggest its critical role in fusion. Lastly, TMEM95, which disappears after the acrosomal reaction, prepares the sperm surface for fusion or exposes other fusogenic proteins necessary to complete the process [22,27]. Our experiments showed that just 90 min of in vitro treatment with a high dose of PS-MPs (210 µg/mL) induces slight but significant downregulation of all six aforementioned genes.

These results are surprising, considering the modest contribution of developing spermatozoa in the context of the whole ejaculates and the mainstream view, although highly debated, of silent transcription/translation of mature sperm cells [67,68,69,70,71]. In this regard, it would be interesting to investigate the molecular mechanisms underlying the observed decreased of the different transcripts, in order to dissect not only the possible contribution of transcription/translation dynamics in a new experimental condition, but also additional control mechanisms affecting mRNA stability (also due to other RNA molecules contained in sperm cells) as a consequence of the PS-MPs exposure. Currently, direct evidence linking a 20–30% reduction in the expression of spermatozoa-specific fusion-related genes to functional impairments in fertilization is limited in the literature. However, even minimal decreases in gene expression, particularly in genes associated with critical cellular processes, warrant further investigation to understand their potential biological implications. Research indicates that small changes in RNA expression, even below the commonly used 2-fold threshold, can be biologically significant [72]. These subtle alterations may reflect underlying biological processes and contribute to pathological mechanisms. This highlights the importance of exploring the role of these variations in male fertility and the mechanisms of microplastic action.

The results, obtained through an in vitro experimental system consisting of cells from ejaculates and brief exposure time, suggest that longer/chronic exposure to PS-MPs can severely compromise various aspects of the fertilization process, from the preparation and stabilization of the mature sperm not properly equipped for fusion (SPACA6, SOF1, TMEM95) to the actual disruption of gamete fusion (IZUMO1, DCST1, and DCST2) leading to difficulties in conception and potentially contributing to male infertility. However, it is important to note that our study investigated the genotoxic effects of microplastics on sperm cells in vitro, allowing us to isolate and examine their direct impact under controlled conditions. A limitation of this approach lies in its inability to fully replicate the complexity of real-world environmental exposures, where factors like indirect exposure, biological barriers, and varying concentrations play a significant role. Ours in vitro studies, focus on controlled conditions to uncover cellular and molecular mechanisms of toxicity. Despite the limitation, our findings provide valuable insights into the potential risks of microplastics on gametes and serve as a basis for future research under more representative conditions.

Furthermore, the identification of gene alterations induced by microplastics could open the door to novel therapeutic or diagnostic approaches for managing fertility issues associated with environmental contaminants.

Monitoring the expression of sperm–oocyte fusion genes, alongside baseline analyses of seminal fluid and evaluation of sperm DNA fragmentation, could help identify individuals at risk of infertility early on and serving as sensitive indicators of environmental contaminants. Analyzing these genes in sperm samples could become a non-invasive diagnostic tool for assessing reproductive health, enabling more accurate diagnoses and timely interventions. Additionally, changes in expression levels could be used to monitor the effectiveness of treatments aimed at mitigating the impact of pollution on fertility.

## Figures and Tables

**Table 1:** Parameters of semen fluid selected for the study. Sperm parameters were expressed as mean ± SD.

SPERM PARAMETERS	Mean ± SD
**Semen volume (mL)**	** 3.5 ± ** **0.5**
**pH**	** 7.2 ± ** **0.3**
**Sperm concentration (×10^6^ sperm/mL)**	** $56\;\times \;10^{6}\;\pm \;$ ** **15.6**
**Viability (%)**	** 86.00 ± ** **4.0**
**Total Motility (%)**	** 83.00 ± ** **3.0**
**Normal morphology**	** 11.60 ± ** **4.0**

**Table 2:** Genes involved in spermatozoon-oocyte fusion. For each gene, the official symbol, official full name, PCR primers, gene type and related organism.

Official Symbol	Official Full Name	PCR Primers	Gene Type	Organism
***DCST1***	*DC-STAMP domain containing 1*	5′-AAGCTGCGTTGCTCCTATGT-3′ 5′-CATGCTTGCGGTCAAACCAA-3′	Protein coding	Homo sapiens
***DCST2***	*DC-STAMP domain containing 2*	5′-GCAACAGCTCCAAGAAGTGC-3′ 5′-GGTGAGAATGCTGACAGGCT-3′	Protein coding	Homo sapiens
***IZUMO1***	*Izumo sperm–oocyte fusion 1*	5′-TTTCACGTCACAGTGTTGCC-3′ 5′-GAGGCTTATGGACGACTCCG-3′	Protein coding	Homo sapiens
***SOF1***	*Sperm−oocyte fusion required 1*	5′-AAAAGTCGAAGCAGGGAGGT-3′ 5′-GCATTAGCTTTCCACAGGCG-3′	Protein coding	Homo sapiens
***SPACA6***	*Sperm acrosome associated 6*	5′-GGCGACCAGGCTATGTTTTC-3′ 5′-GGCATATCTCGGAAATAGGACAAG-3′	Protein coding	Homo sapiens
***TMEM95***	*Transmembrane protein 95*	5′-AAAGTGGCTTGTGAAGACGC-3′ 5′-ACTGTCTTTGGAGCTGGGAC-3′	Protein coding	Homo sapiens
***GAPDH (reference gene)***	*Glyceraldehyde-3-phosphate dehydrogenase*	5′-GAAGGTGAAGGTCGGAGTC-3′ 5′-GAAGATGGTGATGGGATTT-3′	Protein coding	Homo sapiens

**Table 3:** Loss and acquisition of polymorphic bands in sperm DNA following treatment with 105 μg/mL PS-MPs and 210 μg/mL PS-MPs for 30, 60, and 90 min.

Minutes of Exposure	Lost Bands at 105 μg/mL PS-MPs	Lost Bands at 210 μg/mL PS-MPs	Gained Bands at 105 μg/mL PS-MPs	Gained Bands at 210 μg/mL PS-MPs
30′	290 bp	290–350 bp	-	690 bp
60′	500–700–1000 bp	350–500–700–1000–1100 bp	200 bp	-
90′	290–400–550–1100 bp	290–320–700–1000 bp	350 bp	200–300 bp

## Data Availability

The data generated and analyzed during this study are available from the corresponding author upon reasonable request.
